# Mental health literacy in maintenance hemodialysis: a qualitative study on recognition, knowledge fragmentation, and stigma-related help-seeking barriers

**DOI:** 10.3389/fpubh.2026.1951731

**Published:** 2026-09-30

**Authors:** Wenyi Xiao, Chendong Xue, Yingying Che, Long Ma, Hui Yao, Haixia Li

**Affiliations:** 1 Ningxia Hui Autonomous Region People’s Hospital, Yinchuan, China; 2 School of Nursing, Ningxia Medical University, Yinchuan, China

**Keywords:** help-seeking behavior, maintenance hemodialysis, mental health literacy, qualitative research, stigma, thematic analysis

## Abstract

**Background:**

Psychological distress is highly prevalent among patients undergoing maintenance hemodialysis (MHD), yet qualitative evidence on their mental health literacy (MHL) remains scarce.

**Objective:**

To explore the actual level of mental health literacy, existing challenges, and needs for psychological services among patients on maintenance hemodialysis; to identify the constituent dimensions of mental health literacy in this population; and to provide qualitative evidence for developing a localized, specialized mental health literacy scale for this group and implementing targeted psychological interventions.

**Methods:**

Using purposive sampling, semi-structured in-depth interviews were conducted with MHD patients meeting the inclusion criteria at the Hemodialysis Center of a Grade III-A hospital in Ningxia from December 2023 to February 2024. The principle of maximum diversity was applied to ensure sample diversity, and the number of interviews was determined based on data saturation; a total of 11 patients were interviewed. Data were managed using NVivo 14.0 software and analyzed using Braun and Clarke’s thematic analysis method.

**Results:**

A total of 3 core themes and 6 subthemes were identified. Theme 1: Heterogeneity Recognizing of Mental Illness. Theme 2: Fragmented Mental Health Knowledge. This includes: 2a Polarized Approaches to Health Information; 2b Superficial Attribution Patterns; 2c Polarization of Self-Regulation Strategies; 2d Uneven Awareness of Mental Health Resources. Theme 3: Ambivalent attitudes toward mental illness and seeking help. This includes: 3a Cognitive Attitudes: Coexistence of Stigma and Normalization; 3b Behavioral Attitudes: The Gap between Intention and Action.

**Conclusion:**

MHL among MHD patients is characterized by insufficient illness recognition, fragmented knowledge, and pronounced stigma-related help-seeking barriers. Targeted, tiered psychoeducation and a culturally adapted MHL assessment tool are urgently needed for this population.

## Introduction

End-stage renal disease (ESRD) is characterized by irreversibility, lifelong treatment, and a high incidence of complications. Currently, maintenance hemodialysis (MHD) is the primary renal replacement therapy for prolonging the survival of patients with ESRD (1). Long-term, regular dialysis exposes patients to multiple stressors, including persistent physical pain, significant financial burdens, social withdrawal, and changes in family relationships. These factors lead to a high prevalence of negative emotions such as anxiety, depression, irritability, and insomnia. Epidemiological data indicate that the prevalence of psychological disorders among patients on maintenance hemodialysis ranges from 24.75 to 50.56%, which is significantly higher than that observed in the general chronic disease population (2). Adverse psychological states not only reduce patients’ adherence to dialysis treatment and increase the risk of dialysis complications such as hypertension and cardiovascular events, but also severely impair patients’ quality of life and may even increase their risk of suicide (3).

Mental health literacy refers to an individual’s comprehensive ability to identify psychological issues in themselves and others, acquire knowledge related to mental health, master methods of emotional regulation, and develop a positive attitude toward seeking psychological help; it serves as the core foundation for individuals to practice self-care and proactively address psychological crises (4). Enhancing mental health literacy among patients with chronic diseases can effectively facilitate the early identification of potential psychological risks, promote the proactive management of negative emotions, and eliminate the stigma associated with seeking psychological help, thereby reducing the incidence of mental disorders at the source (5). Currently, domestic research on the mental health literacy of patients with chronic diseases has largely focused on those with diabetes, stroke, and cancer, with few specialized studies targeting individuals on hemodialysis. Furthermore, existing general mental health literacy scales do not account for disease-specific characteristics of dialysis patients—such as lifelong dialysis, persistent physical discomfort, and significant social discrimination—and are therefore unable to accurately assess the true level of mental health literacy within this population (6, 7).

Research on mental health literacy among patients with kidney disease began relatively early in other countries. Studies have confirmed that dialysis patients generally suffer from a lack of psychological knowledge and a low willingness to seek help, and that their stigma associated with mental illness is significantly higher than that of patients with other chronic diseases (8). Most existing domestic studies rely on quantitative questionnaire surveys to analyze the prevalence of anxiety and depression among dialysis patients (9). Still, they lack in-depth interviews grounded in patients’ genuine subjective experiences. Consequently, these studies are unable to uncover patients’ hidden psychological concerns, challenges in accessing information, and barriers to seeking help.

Based on the three-dimensional theoretical framework of mental health literacy and considering the local healthcare environment, family characteristics, and sociocultural background of patients with MHD in the Northwest region, this study thoroughly explores the actual dimensions of mental health literacy within this population. It identifies patients’ cognitive gaps and barriers to seeking help, thereby providing an evidence-based foundation for healthcare professionals at hemodialysis centers to develop mental health education programs and psychological support measures tailored to patients’ needs. This approach aims to enhance dialysis patients’ ability to manage their own mental health and improve their long-term prognosis and quality of life.

## Methods

### Study design and setting

This was a qualitative study employing semi-structured, in-depth individual interviews. Data were collected at the Hemodialysis Center of a tertiary (Grade III-A) hospital in Ningxia, China, between December 2023 and February 2024. The study followed the Consolidated Criteria for Reporting Qualitative Research (COREQ) checklist (10). The research team included five members with nursing and qualitative research backgrounds: WX (Master’s candidate, 2 years of hemodialysis experience, trained in qualitative methods) and CX (Master’s candidate, trained in qualitative methods) conducted all interviews; WX and YC performed independent coding under the supervision of HY and HL (professors with 10–15 years of qualitative research experience). Before data collection, the team documented potential biases—e.g. before data collection, the research team engaged in regular discussion meetings to acknowledge potential biases arising from their clinical and academic backgrounds. These discussions were documented in meeting minutes and revisited during data analysis to ensure analytical rigor.

### Sampling and participants

Using a combination of purposive sampling and maximum diversity sampling, respondents were selected based on criteria such as age, gender, educational level, duration of dialysis, household income, marital status, and place of residence to ensure the richness and representativeness of the interview data and to avoid research bias resulting from sample homogeneity.

#### Inclusion criteria

① Meets the diagnostic criteria for end-stage renal failure as outlined in the Guidelines for the Screening, Diagnosis, and Prevention and Treatment of Chronic Kidney Disease and has been undergoing regular hemodialysis for ≥3 months; ② Fixed dialysis regimen: 2-3 dialysis sessions per week, each lasting 3-4 h; age ≥18 years; ③ Informed consent; voluntary participation in this interview.

#### Exclusion criteria

① Patients currently undergoing combined peritoneal dialysis; ② Patients with severe physical functional impairments, such as serious cardiovascular or cerebrovascular diseases or physical disabilities, who are unable to cooperate with the interviewer; ③ Patients who have previously undergone a kidney transplant or are scheduled to undergo a kidney transplant in the near future; ④ Patients with a history of diagnosed primary mental illness or cognitive impairment.

### Sample size

The sample size was determined in accordance with the principle of data saturation in qualitative research. Data saturation was deemed to have been reached—and interviews were terminated—when three consecutive interviews yielded no new perspectives, codes, or themes. This study ultimately interviewed 11 patients undergoing maintenance hemodialysis, and the data were fully saturated. All eligible patients who were invited agreed to participate; no patients declined or withdrew prior to completion.

### Research tools

#### General information questionnaire

Developed by the research team, this questionnaire includes demographic data (age, gender, marital status, educational level, occupation, type of health insurance, place of residence, and living arrangement), household economic data (monthly per capita household income), and dialysis-related data (duration of dialysis), and is designed to comprehensively collect baseline information from respondents.

#### Semi-structured interview guide

Using the three-component structure of mental health literacy as the core framework, a preliminary interview guide was drafted following a review of relevant domestic and international literature and discussions within the research team. Preliminary interviews were conducted with two patients, and the wording of the guide was optimized based on the patients’ level of understanding and the logical flow of the questions. This process resulted in the final interview guide, as detailed in Table 1.

**Table 1:** Semi-structured interview guide.

Number	Interview questions
1	Since you began dialysis after being diagnosed with kidney disease, how has your overall emotional state changed? Can you identify what type of psychological issue you are experiencing?
2	When you are feeling down or experiencing negative emotions, what specific physical and behavioral signs do you exhibit?
3	What do you think are the main causes of these psychological issues? How have these negative emotions affected your life and your dialysis treatment?
4	What sources do you usually use to learn about emotional regulation and mental health conditions?
5	When you cannot relieve negative emotions on your own, what do you usually do to deal with them?
6	Where did you learn the methods for emotional regulation and psychological counseling that you know?
7	If your psychological distress becomes more severe, would you be willing to seek help from professionals such as psychologists or healthcare providers?
8	Do you know any dialysis patients who are struggling emotionally? How do you view the psychological issues these patients face? How would you go about helping them?

### Data collection methods

Interviews were conducted in a dedicated conference room at the Hemodialysis Center, which provided a quiet and private setting. Interview times were scheduled to avoid dialysis treatment periods, and each interview lasted between 30 and 45 min. Prior to the interview, participants were provided with a detailed explanation of the study’s purpose, procedure, potential risks, and their right to voluntarily withdraw, and they signed a written informed consent form. The interviewers had received systematic training in qualitative research and introduced themselves before the interview began to establish rapport. The interviewers (WX and CX) had received systematic training in qualitative research and introduced their names, backgrounds, and roles at the beginning of each interview; they also explicitly stated that they were not involved in the participants’ clinical care in order to minimize power imbalances. No one else was present during the interviews except for the participants and the interviewers. The interviews strictly followed the outline, while the order of questions was flexibly adjusted and follow-up questions were asked based on the actual conversational context. The researchers maintained a neutral stance, avoided leading questions, and encouraged participants to fully express their genuine feelings. The entire interview was audio-recorded, and nonverbal cues and on-site notes were recorded simultaneously. When no new information emerged from the interview, data saturation was deemed to have been reached, and the interview was terminated. Transcriptions were completed within 24 h of the conclusion of each interview; the transcription process retained fillers, pauses, and markers of emotional expression to enhance the richness of the text.

### Data analysis

Text data were organized using NVivo 14.0 software. Two researchers (WX and YC) independently coded the first three transcripts, then compared their results and resolved any discrepancies through discussions with HY and HL. Subsequently, the entire research team (WX, CX, YC, LM) organized the coding into themes and, through consensus reached during a team meeting, finalized the definitions and names of the themes. Throughout the analysis process, the team held regular discussions to ensure transparency and rigor in the analysis. Analysis was conducted using the thematic analysis method proposed by Braun and Clarke (11): ① Familiarization with the data: Transcribing audio data and repeatedly and actively reading the transcribed interview data; ② Generating initial codes: The researcher began noting potential data related to the research themes, questions, connections between data items, and other preliminary ideas; ③ Identifying themes: Examining the coded and organized data extracts to identify potential themes with broader significance; ④ Reviewing themes: This involves a two-level analysis process. In the first level, the researcher reviews the coded data assigned to each theme to ensure appropriate matching; in the second level, the researcher determines whether individual themes align meaningfully with the dataset and adequately represent the entire data corpus; ⑤ Defining and Naming Themes: Create definitions and narrative descriptions for each theme, including why it is important to the broader research question, and review the theme names to ensure they are concise and adequately descriptive; ⑥ Writing the Manuscript: Draft the final description of the analysis and findings.

### Assurance of reliability

The following measures were taken in accordance with Lincoln and Guba’s criteria for reliability: Reliability: Researcher triangulation and participant validation were employed; Generalizability: Detailed contextual descriptions and participant profiles were provided to help readers assess the applicability of the findings; Dependability: A comprehensive audit trail was established that details sampling decisions, the coding process, and the evolution of themes; Verifiability: Regular team discussions were held to acknowledge personal assumptions and biases, thereby reducing subjective bias. Member checking was conducted with three participants (P2, P7, P10) to verify transcript accuracy and preliminary findings.

## Results

### Demographic characteristics of respondents

A total of 11 patients were interviewed, including 5 men and 6 women, aged 20–62 (45.45 ± 14.57) years, with a dialysis duration of 1–10 (4.36 ± 2.98) years. The respondents were distributed across various dimensions, including age, gender, educational level, marital status, and duration of dialysis, demonstrating good sample heterogeneity. See Table 2 for details.

**Table 2:** Demographic characteristics of interviewed patients (*n* = 11).

Interviewee ID	Age	Gender	Monthly per capita household income	Educational background	Duration of dialysis	Marital status	Occupation	Health insurance	Place of residence	Housing situation
P1	36	Male	4,000~	High school	10	Unmarried	Currently employed	Employee health insurance	Towns and cities	With my family
P2	48	Female	4,000~	Undergraduate	3	Married	Currently employed	Employee health insurance	Towns and cities	With my family
P3	37	Female	<2000	High school	2	Divorce	None	Resident health insurance	Towns and cities	Myself
P4	30	Female	4,000~	Undergraduate	1	Married	Currently employed	Employee health insurance	Towns and cities	With my family
P5	58	Female	<2000	Junior high school	6	Married	None	Resident health insurance	Rural areas	With my family
P6	60	Female	<2000	Junior high school	7	Married	None	Resident health insurance	Rural areas	Myself
P7	59	Male	<2000	High school	8	Married	Retirement	Employee health insurance	Towns and cities	With my family
P8	62	Male	<2000	Junior high school	1	Married	None	Resident health insurance	Towns and cities	With my family
P9	20	Male	0	Junior high school	3	Unmarried	None	Resident health insurance	Rural areas	With my family
P10	56	Male	2000~	High school	4	Married	Currently employed	Employee health insurance	Towns and cities	With my family
P11	34	Female	4,000~	Undergraduate	3	Unmarried	Currently employed	Employee health insurance	Rural areas	With my family

### Results of theme extraction

Through systematic coding and thematic analysis of the interview data, this study—using O’Connor et al.’s simplified three-dimensional model of mental health literacy as its theoretical framework—extracted three themes and six subthemes from the interview data of patients with MHD, as shown in Table 3 and Figure 1.

**Table 3:** Results of the extraction of themes and subthemes related to mental health literacy among patients with MHD.

Theme	Sub-theme
1. Heterogeneity recognizing of mental illness	—
2. Fragmented mental health knowledge	2a. Polarized approaches to health information
	2b. Superficial attribution patterns
	2c. Polarization of self-regulation strategies
	2d. Uneven awareness of mental health resources
3. Ambivalent attitudes toward mental illness and seeking help	3a. Cognitive attitudes: coexistence of stigma and normalization
	3b. Behavioral attitudes: the gap between intention and action

**Figure 1:**
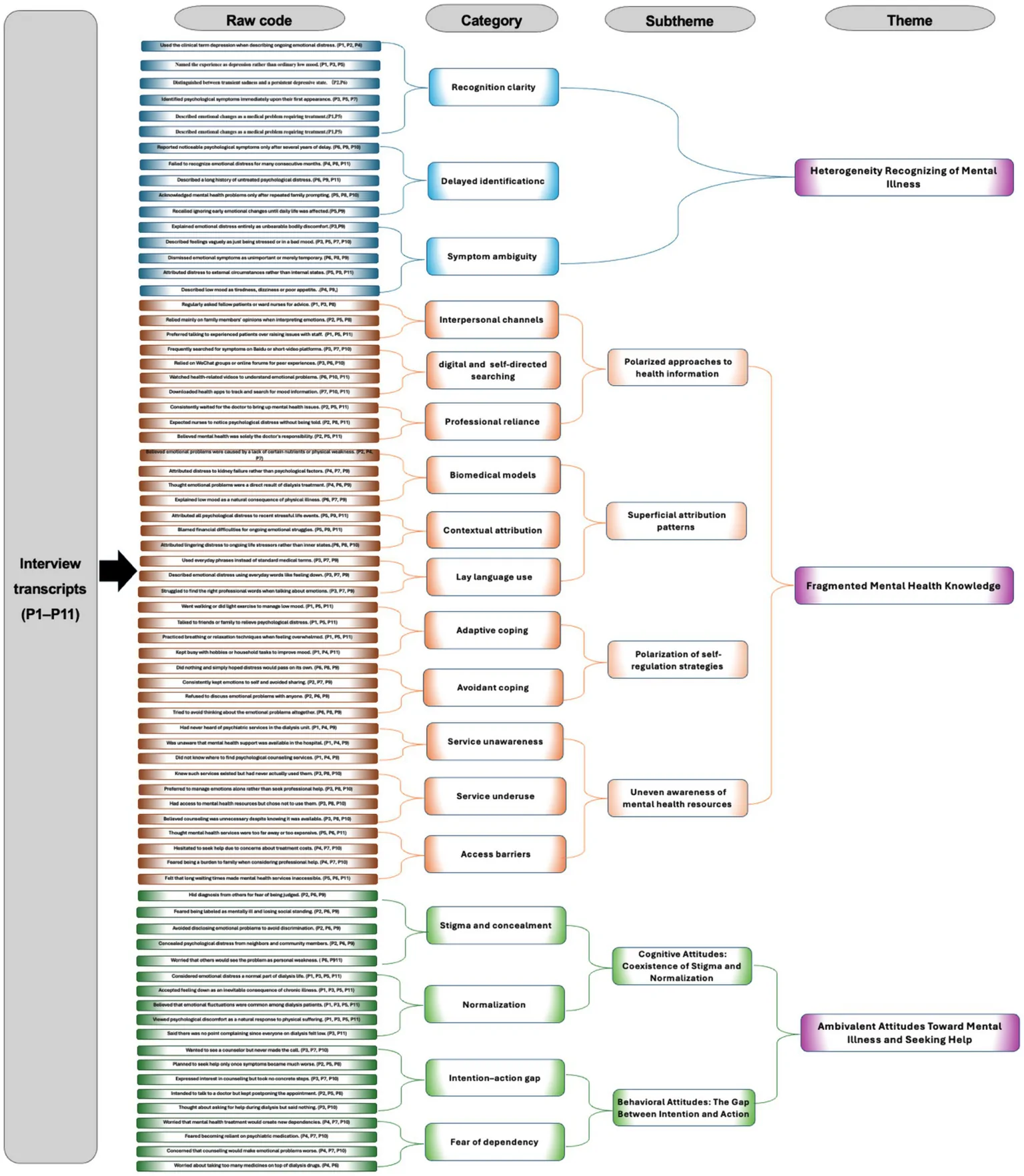
Thematic map of mental health literacy among maintenance hemodialysis patients.

### Theme 1: heterogeneity recognizing of mental illness

This theme refers to whether patients can perceive the characteristics of mental illness or accurately identify the type of mental illness they have. The interviews revealed that MHD patients exhibit significant individual differences in this regard.

Four participants (P1, P2, P4, P11) were able to associate their subjective distress with specific concepts of mental disorders, demonstrating a high level of recognition ability. For example, P1 clearly described the depressive symptoms they had experienced and their severity:

*“Back then, I felt like I might have been experiencing mild depression—I’d had those thoughts before, you know, thoughts of taking my own life…I just felt like life was meaningless and didn’t want to do anything.” (P1, male, 36)*

However, although six participants (P3, P5, P7, P8, P9, P10) can perceive emotional abnormalities, they are unable to categorize them as a specific, identifiable mental disorder and can only express their distress using vague, everyday language. P7’s description typifies this state of having the experience but no label:

*“I just don’t feel very well. It's starting to affect my life. I’m feeling pretty scattered—every day is a mess, I snap at people for no reason, and I’m in a really bad mood. I don’t even know what’s wrong with me.” (P7, male, 59)*

One participant (P6) reported severe insomnia and appetite loss while explicitly rejecting the description of being psychologically distressed, attributing both to the illness. This case matters because it shows that the recognition deficit in this population is not simply a matter of missing vocabulary: for at least one participant, the somatic presentation actively displaced the psychological one.

*“My mood is fine—I don’t have times when I’m feeling down. It’s just that I can’t sleep; for the first half of the night I simply can’t get to sleep at all.” (P6, female, 60)*

Recognition, where present, was also delayed. P10 described recognizing the problem only after it had become severe:

*“At first I didn’t. Later I felt it wasn’t working—you can clearly tell your whole state is wrong, that it’s serious—and then I thought, I have to deal with this, or it will wreck my life and my dialysis.” (P10, male, 56)*

It is worth noting that even among patients who can recognize their symptoms, this recognition often occurs only after they have been experiencing emotional distress for a considerable period of time; early recognition is generally lacking. This heterogeneity in recognition ability means that even if patients subjectively feel distress, they may find it difficult to seek help unless they have the ability to translate that distress into medical terms.

### Theme 2: fragmented mental health knowledge

The four subthemes below are not four separate deficits. They map onto the four elements of the knowledge component of mental health literacy as originally defined: knowing how to seek mental health information, knowledge of risk factors and causes, knowledge of self-treatments, and knowledge of professional help available. Read in sequence, they form a closed loop. It is this loop, rather than any single gap in knowledge, that prevents distress in this population from reaching services.

#### Theme 2a: polarized approaches to health information

The interviews revealed a marked polarization in patients’ approaches to obtaining information. Six participants (P2, P3, P4, P9, P10, P11) demonstrated a tendency to actively seek out information, using the internet and social media to conduct their own research. P10 described their information-seeking behavior as follows:

*“I do my own research—I just look things up online on my phone, watch short videos, or search on Baidu.” (P10, male, 56)*

P11, on the other hand, makes comprehensive use of a variety of channels, including online platforms, WeChat official accounts, and personal networks:

*“Yeah, I look things up online myself sometimes. I’ll check out posts on that WeChat official account now and then…And my friends also tell me about things and recommend books to me.” (P11, female, 34)*

P3, despite the lowest income tier apart from P9, also searched actively, but privately, to avoid burdening her son:

*“Most of the time I look it up online…I search on Baidu or on short-video platforms and read a bit myself. I don’t really talk to my son about it, because I think it would just add to his burden.” (P3, female, 37)*

However, the remaining five (P1, P5, P6, P7, P8) were passive recipients who could not identify any information source beyond sporadic clinical encounters. P5’s statement reveals this passivity:

*“The nurses never told me about it, so I don’t really know—I never thought in that direction.” (P5, female, 58)*

P6 and P8, when asked where they would turn for psychological knowledge, could name only a clinician or their own reflection:

*“I wouldn’t know—I’d ask a doctor, or else just sit and think it through by myself. Once I figure it out, it should be fine.” (P6, female, 60)*

*“I haven’t thought about it. Though I might ask a doctor.” (P8, male, 62)*

The polarization did not map cleanly onto education. The five passive recipients were the four oldest participants (P5, 58; P6, 60; P7, 59; P8, 62) plus P1, who was well resourced but obtained his information from clinicians and personal experience rather than by searching. Conversely, P9, aged 20 with junior high schooling and no income, was an active seeker. Age and habitual digital use therefore appeared more discriminating than years of schooling.

What matters more than who searched is what the searching produced. None of the six active seekers described encountering information that changed their understanding of what was wrong or of what could be done about it. P2 acquired the word “depression” from short videos and continued to manage alone; P10 learned from short videos that exercise might improve his mood, which is a self-management technique rather than a route to care; P11 searched across several platforms and assembled a regimen of exercise, short videos, and social contact that she credited to her own efforts and to a nurse’s suggestion. Active searching therefore enlarged the volume of information without altering its structure. The explanation remained that distress is a consequence of dialysis to be absorbed, and in this respect the active and passive participants arrived at the same place by different routes.

#### Theme 2b: superficial attribution patterns

Patients’ understanding of the causes of psychological distress primarily centers on three types of factors: the impact of the illness itself, financial pressure, and a lack of social support. P5 attributes emotional fluctuations to difficulties in coping with the illness and a perceived lack of family support:

*“First, I suddenly had to start dialysis, and I’m having a hard time coming to terms with that reality; second, the people around me don’t seem to care much about me—neither my children nor my spouse are very concerned about me.” (P5, female, 58)*

P3, on the other hand, explained his emotional state from the perspective of multiple losses:

*“I have this illness, I'm divorced, and my son is in college. We rely on his father for financial support, and I feel like a total failure.” (P3, female, 37)*

P10 mentions the influence of physiological mechanisms, demonstrating a partial understanding of the bio-psychological connection:

*“I have trouble sleeping at night and have no appetite. The more I struggle to fall asleep, the more irritable I get, which affects my mood. When I don't sleep well, my mental state suffers, and that creates even more psychological problems.” (P10, male, 56)*

However, participants generally lacked a systematic understanding of risk factors, and almost none mentioned deeper psychological factors such as personality traits, coping strategies, and the quality of social support. This limited understanding may make it difficult for them to address prevention and early intervention.

#### Theme 2c: polarization of self-regulation strategies

When faced with negative emotions, patients exhibit two distinctly different coping styles. Four participants (P1, P5, P10, P11) adopt proactive coping strategies to alleviate their distress through distraction and behavioral activation. P5 describes his distraction techniques:

*“Listen to some music, watch videos on TikTok, take a stroll in the park, get some exercise, or chat with your kids.” (P5, female, 58)*

P11, through personal experience, demonstrates the value of active participation in alleviating anxiety.

*“The nurse recommended that I exercise, so I started playing badminton with people in my neighborhood every day. When I’m busy, I don’t dwell on things as much, and I feel less anxious.” (P11, female, 34)*

However, four participants (P3, P6, P7, P8) adopt a passive-endurance strategy, viewing their emotional distress as an unchangeable part of life. P3 expressed this habitual coping logic:

*“At first, I thought this illness was incurable, but as I continued with dialysis, my outlook gradually improved. I just came to terms with it, and over time, I got used to the negative emotions—there’s no point in worrying about them.” (P3, female, 37)*

P7, on the other hand, pins its hopes on time naturally resolving the issue:

*“There’s nothing to worry about—it'll get better with time. Just don't think about it or let your mind wander, and it'll be fine.” (P7, male, 59)*

The remaining three (P2, P4, P9) combined elements of both: they used distraction and self-persuasion but described the effect as partial and temporary. P2, who wanted to exercise, was constrained by her physical condition and reverted to solitary absorption:

*“I used to love exercise, and it did ease my mood, but now that I have this illness I can’t do much intense activity, and I don’t want to talk to others—I just try to absorb it myself. But absorbing it like that, over time, leaves me feeling somewhat depressed.” (P2, female, 48)*

Although this passive coping strategy may maintain superficial stability in the short term, in the long run it may contribute to the chronicity of psychological symptoms and indirectly affect treatment adherence.

#### Theme 2d: uneven awareness of mental health resources

When asked about available professional mental health resources, patients mentioned only a very limited range of options. Only 4/11 participants (P2, P4, P10, P11) could name a specific resource and endorse it. P2 identified psychiatric outpatient clinics:

*“Some hospitals have specialized psychiatric outpatient clinics—you could go to one of those, I suppose.” (P2, female, 48)*

P10 and P11 endorsed help-seeking as ordinary healthcare behavior:

*“I’d give it a try. I think seeing a psychologist is perfectly fine, perfectly normal—just like seeing a doctor when you’re sick. I’ve always thought of it as a normal thing to do.” (P10, male, 56)*

*“I think I would. I don’t think there’s anything to be ashamed of in seeing a psychologist—if you have a problem, going to get it resolved is perfectly normal.” (P11, female, 34)*

The remaining 7/11 (P1, P3, P5, P6, P7, P8, P9) either could not name any resource or actively dismissed its relevance. However, the responses from P7 and P9 revealed deeper cognitive blind spots:

*“You can just work through psychological issues on your own—you don’t need to see a doctor or take medication, right? It’s not a big deal.” (P7, male, 59)*

*“Professional help? I don’t really know much about that. Do you really need to see a doctor for this? I think it’s just a mental issue—you have to figure it out on your own. A doctor can’t really help much.” (P9, male, 20)*

Furthermore, none of the patients mentioned diverse resources such as mental health support groups, peer support, online mental health platforms, or social work services. This narrow perspective significantly limits patients’ ability to access effective help when they need it. Based on the demographic data presented in Table 2, patients with limited access to professional mental health resources predominantly shared the following characteristics: lower educational attainment (junior high school or below, e.g., P5, P6, P8, P9), lower monthly per capita household income (<2000 CNY, e.g., P3, P5, P6, P8, P9), rural residence (P6, P9), unemployment or being out of the labor force (P3, P5, P6, P8, P9), and reliance on resident health insurance rather than employee health insurance (P3, P5, P6, P8, P9). Notably, these patients also tended to be older (e.g., P5, P6, P8 in their late 50s to early 60s). In contrast, patients with higher education (undergraduate or high school), urban residence, and employee health insurance (e.g., P1, P2, P4, P10, P11) demonstrated relatively broader awareness of mental health resources, although their knowledge remained incomplete.

### Theme 3: ambivalent attitudes toward mental illness and seeking help

#### Theme 3a: cognitive attitudes: coexistence of stigma and normalization

Six participants (P3, P4, P5, P7, P9, P11) expressed stigma, but it was attached to disclosure rather than to the distress itself. P3 and P9 both focused on being known by others:

*“This illness itself is already something I find hard to talk about, and on top of that, I have mental health issues—people are bound to judge me and make fun of me.” (P3, female, 37)*

*“If others find out, they’ll just laugh at you. They’ll think, ‘Oh, he’s sick again and has mental health issues.’ I can’t stand that kind of look.” (P9, male, 20)*

P5 anticipated ridicule and linked it to the devaluation already carried by dialysis:

*“I don’t usually tell people—what’s the point? They can’t help you, and they might laugh at you. It’s not a good illness, it can’t be cured, and you become a useless person.” (P5, female, 58)*

This sense of shame is particularly pronounced in the group therapy setting of dialysis. In the relatively enclosed, long-term treatment environment of a hemodialysis center, fellow patients and their families constitute the patient’s primary social circle; therefore, the pressure of negative judgment from this group significantly inhibits the patient’s willingness to seek help. Patients spend extended periods in semi-public shared spaces where privacy boundaries are blurred, further exacerbating their fear of exposure.

On the other hand, five participants (P4, P7, P8, P10, P11) view mental illness as a normal phenomenon no different from physical illness and adopt a stance of destigmatization. P7’s statement reflects this relatively healthy cognitive framework:

*“Everyone has times when they’re feeling down. Mental illness is just like any other illness—so what? Besides, we’re already on dialysis—isn’t it even more normal to have some mental health issues?” (P7, male, 59)*

This difference in attitude suggests that destigmatization education may yield positive results among people with MHD, provided that an appropriate approach is identified.

#### Theme 3b: behavioral attitudes: the gap between intention and action

Participants also showed divergent attitudes regarding their willingness to seek help. P10 represented a proactive stance toward seeking help:

*“I would go see one. I think seeing a psychologist is perfectly normal—just like seeing a doctor when you’re sick. I’ve always thought of it as a normal thing to do.” (P10, male, 56)*

However, P3’s response revealed that shame acts as a significant barrier to seeking help:

*“There’s this feeling that’s hard to put into words—I feel ashamed about seeing a therapist. What if people found out that so-and-so was seeing a therapist? They’d definitely start gossiping and making fun of me, and I just can’t handle that.” (P3, female, 37)*

It is worth noting that even among participants who expressed a positive attitude, there remains a significant gap between their intentions and their actual actions. Although P10 claimed to be willing to seek medical care, they admitted to never having actually taken that step, citing uncertainty about which department to visit and fear of being seen by acquaintances as the reasons. This indicates that a positive attitude alone is insufficient to prompt the act of seeking help; practical constraints—such as unclear medical procedures, concerns about costs, and transportation difficulties—are equally significant barriers.

## Discussion

### Recognition heterogeneity and delayed identification of mental illness

The results of this study indicate that patients with MHD exhibit significant heterogeneity in their ability to identify mental disorders. Although 4/11 patients can correlate their subjective experiences of distress with specific concepts of mental disorders—and can even clearly describe a depressive state accompanied by negative thoughts—the vast majority of patients find themselves in a cognitive dilemma where they experience symptoms but lack the labels to describe them. They are able to perceive emotional abnormalities and their impact on daily functioning but are unable to classify these as recognizable medical problems. This disconnect between emotional perception and pathological cognition is not merely a lack of knowledge but reflects the patients’ lack of a cognitive framework for conceptualizing and medicalizing their subjective experiences of distress (12).

It is worth noting that even among patients with some ability to recognize their symptoms, symptom recognition often lags behind the actual course of the illness; patients typically do not realize they may have a mental health problem until their emotional distress has persisted for several months or even longer (13). This delayed recognition is particularly pronounced among the MHD population. Dialysis treatment requires patients to adhere to strict fluid and dietary restrictions over the long term and to travel to and from the hospital regularly for treatment. Any deterioration in mental health at any stage can directly translate into a risk to survival by reducing treatment adherence; therefore, delayed recognition actually constitutes a latent threat to patients’ long-term prognosis (14). The findings of this study explain, at the mechanistic level, the paradox observed in previous quantitative studies—namely, the coexistence of high screening positivity rates and low clinical diagnosis rates. Epidemiological data indicate that the prevalence of anxiety and depression among MHD patients ranges from 24.75 to 50.56%, yet the proportion of patients who actively seek psychological help remains extremely low (2, 15, 16).

This disparity may be partly because psychological symptoms—such as fatigue, insomnia, and loss of appetite—overlap significantly with the physical adverse effects of dialysis treatment and the progression of kidney disease itself. Patients tend to attribute these manifestations to side effects of dialysis or the worsening of their physical illness, thereby obscuring the nature of their psychological problems (10, 17). This confusion in attribution objectively exacerbates cognitive barriers at the recognition level, making it difficult for patients to distinguish psychological symptoms from the complex spectrum of physical symptoms. More critically, for dialysis patients with lower educational levels whose attention has long been dominated by physical symptoms, this is not a spontaneous cognitive shift but rather requires more targeted cognitive interventions.

From the theoretical framework of mental health literacy, recognition ability serves as the starting point of the entire literacy structure; the stronger an individual’s ability to recognize mental health conditions, the more proactive their willingness to seek help and the more effective their self-management will be (4). Therefore, enhancing MHD patients’ ability to recognize mental health issues should be the primary entry point for clinical psychological interventions and one of the core indicators for evaluating the effectiveness of such interventions. These findings suggest that brief, dialysis-specific psychological assessment tools should be integrated into routine, proactive screening prior to dialysis. By helping patients recognize emotional distress as an identifiable health issue rather than an inevitable source of suffering, such screening can bridge the gap between subjective experience and medical conceptualization, thereby promoting earlier self-management behaviors and reducing delays in identification.

### Fragmented mental health knowledge and structural disparities

In terms of knowledge, this study found that MHD patients’ knowledge related to mental health is fragmented. Regarding information sources, patients exhibited a distinct polarization, reflecting class disparities in digital health literacy (18). The 6/11 participants who actively sought information proactively utilized diverse channels—such as the internet, social media, and personal networks—to access information, demonstrating strong self-management capabilities; in contrast, the remaining 5/11 were entirely passive recipients, relying solely on sporadic verbal information from healthcare providers and possessing extremely limited knowledge of professional mental health support resources. Only 4/11 could name and endorse a professional resource, 7/11 could name none or actively dismissed the idea, and almost no one was aware of more diverse psychological support resources such as support groups, online counseling platforms, and cognitive behavioral therapy (19). This polarization suggests that the traditional model of uniformly distributing educational brochures may only reach those 6/11 participants who already possess the ability to actively seek information, while patients with low educational attainment, low income, and rural household registration—who are truly isolated from information—are further marginalized (20). Consistent with our demographic data, these marginalized patients were predominantly those with junior high school education or below, monthly incomes below 2000 CNY, rural residency, unemployment, and coverage by resident (rather than employee) health insurance. Given the proportion of these 5/11 participants in the sample of this study, this finding serves as an important warning for educational practices at primary-level hemodialysis centers in the Northwest region.

At the same time, patients’ understanding of the causes of psychological problems is generally limited to superficial factors; they primarily attribute emotional issues to external factors such as the impact of illness, economic pressure, and lack of family support, with almost no one mentioning deeper psychological mechanisms such as personality traits, coping strategies, or cognitive patterns (21). This cognitive limitation leads patients to view psychological problems as direct products of their external environment, rather than as a process that can be actively managed by adjusting their cognitive and coping strategies. Theoretically, this can be explained by Leventhal’s common-sense self-regulation model, in which patients form a certain illness schema where dialysis, financial stress, and insufficient social support are perceived as powerful, external threats, while their personal control over emotional responses is underestimated (13, 22). When psychological distress is viewed as an inevitable consequence of the external environment, patients are more likely to adopt external attributions and passive coping strategies rather than recognizing that their cognitive and coping strategies are modifiable (23). Among patients with chronic illnesses, stronger beliefs about the inevitability of consequences and weaker beliefs in personal control are more likely to lead to avoidance, compliance, and poor psychological adaptability; whereas problem-focused coping, cognitive reappraisal, and behavioral activation can improve outcomes related to psychological distress (22, 24). This pattern is particularly relevant for MHD patients facing lifelong treatment, overlapping physical symptoms, and limited social circles: while they do bear real external burdens, they gradually come to believe that “nothing within me can change how I feel.” Therefore, the cognitive limitations identified in this study are not merely a “lack of knowledge,” but rather a misattribution of controllability. This is precisely why psychoeducation should not only provide information but also train patients to reattribute their psychological distress.

Regarding self-regulation strategies, this study observed that 4/11 patients were able to actively regulate their emotions through methods such as distraction, physical activity, and social interaction, which is consistent with the theory of behavioral activation in positive psychology; however, a further 4/11 adopted a passive coping model of acceptance–endurance–habituation, viewing emotional distress as an inherent part of life on dialysis without seeking intervention. Although this habitual helplessness may maintain superficial psychological stability in the short term, in the long run, it may contribute to the chronicity of psychological symptoms (25) and indirectly affect treatment adherence by undermining self-efficacy. More critically, these patients often do not perceive themselves as needing help, thereby becoming an underserved group in mental health services. These dual limitations in knowledge acquisition and cognitive structure have deep-rooted causes. On the one hand, as reported by patients, MHD patients primarily interact with nephrology healthcare professionals during dialysis; due to their busy clinical schedules, these professionals typically devote most of their energy to adjusting dialysis regimens and managing complications, with relatively insufficient attention paid to patients’ mental health issues or connecting them with relevant resources. This patient-reported structural barrier limits opportunities for timely mental health screening and referral. To address this, hemodialysis centers should integrate brief mental health assessments into routine nursing assessments and establish standardized referral pathways to psychiatric or psychological services, thereby reducing reliance on patient-initiated help-seeking. On the other hand, patients themselves face a narrowing of information access channels due to reduced social participation resulting from their long-term illness, further exacerbating structural deficiencies in mental health literacy.

To address the issue of fragmented knowledge, tiered psychological education should be implemented. For patients with strong digital literacy, interactive online platforms and short video series can be used to deepen their understanding of psychological risk factors and broaden their awareness of diverse professional resources. For passive patients with low health literacy, dialysis nurses should use simplified brochures, pictograms, and peer narratives to conduct face-to-face education, thereby reducing information asymmetry. Crucially, educational content must extend beyond symptom management to include behavioral activation techniques—such as distraction and physical activity—thereby directly enriching patients’ repertoire of self-regulation strategies. Given the observed polarization in help-seeking intentions and actions, motivational interviewing may be particularly valuable for patients who express willingness but hesitate due to practical barriers (e.g., P10), as it can strengthen intrinsic motivation and resolve ambivalence toward seeking professional help.

### Stigma and normalization in help-seeking attitudes

The most central finding of this study regarding the attitude dimension is the conflict between the stigma associated with their illness and the willingness to seek help among patients with MHD. This finding is highly consistent with existing research. Numerous studies have indicated that stigma associated with mental illness is the primary barrier preventing patients from seeking professional help (26, 27). A strong sense of illness-related stigma causes most patients to go to great lengths to conceal their mental health issues, fearing that the negative label of having mental health problems will compound the social discrimination they already face due to dialysis. This fear is further intensified by the unique spatial structure of hemodialysis centers. As relatively closed, semi-public, and long-term collective treatment settings, these centers foster close social networks among patients, between patients and healthcare providers, and among patients’ families, resulting in highly blurred boundaries of privacy. In this environment, simply being seen is a risk in itself, let alone actively revealing psychological vulnerability.

It is worth noting that this sense of stigma exhibits characteristics of dual stigmatization: MHD patients must not only face the physical and social functional impairments caused by end-stage renal disease itself but also endure the social prejudice that may arise from being labeled as dialysis patients; if the label of psychological problems is added to this, the pressure of stigma exhibits a significant cumulative effect. In the Chinese cultural context, mental illness is often equated with mental abnormality, and its impact on the reputation of both the individual and the family is particularly profound. This sociocultural pressure mechanism leads patients to tend toward silence and concealment when facing psychological distress, rather than actively seeking help. On the other hand, 5/11 patients view mental health issues as commonplace phenomena no different from physical illnesses and adopt a distinctly destigmatizing attitude. The formation of this attitude may be related to a process of redefinition resulting from long-term illness; when dialysis itself has become a normal part of life, the accompanying psychological distress no longer carries a particular sense of shame. This link between normalization and destigmatization is achieved through a process of cognitive restructuring: by defining psychological distress as a common and predictable complication of dialysis—rather than an abnormal or morally compromising condition—patients effectively strip the experience of its stigmatizing connotations. In other words, normalization serves to eliminate stigma by replacing the label of “abnormality” with that of “normality,” thereby alleviating concerns about self-image and social standing—concerns that drive patients to conceal and avoid discussing their condition (28–30). Treating mental distress as no different from other illnesses draws on normalization strategies aimed at eliminating stigma; these strategies work to blur the “us versus them” boundaries that perpetuate stigma by emphasizing shared human experiences (30). Within chronic illness populations, Leventhal’s common-sense model further supports this: when patients represent distress as a controllable, illness-linked experience rather than a discrediting personal defect, they are more likely to adopt adaptive coping and less likely to internalize stigma (22). This finding suggests that destigmatization education is not ineffective among the MHD population; normalizing psychological issues rather than treating them as exceptional may be a viable path to reducing the stigma associated with the condition.

The research findings provide actionable insights for eliminating the stigma surrounding mental health education among this population. First, mental health education should adopt a normalizing narrative framework, explicitly presenting psychological distress as a common complication of chronic dialysis rather than a unique or stigmatizing condition. This approach aligns with the cognitive restructuring already demonstrated by P7 and P10, who view mental illness as no different from other medical conditions. Second, because patients worry about being watched or gossiped about by others in the semi-public dialysis setting, destigmatization efforts must be integrated into routine dialysis care while avoiding unnecessary attention; for example, brief mental health education sessions could be incorporated into pre-dialysis assessments or conducted as one-on-one, face-to-face consultations in private counseling rooms, rather than in the form of group lectures. Third, leveraging peer influence is crucial: patients like P11, who already view seeking mental health help as the norm, can be trained as peer educators to share their experiences in a way that patients can relate to, thereby reducing patients’ perception of stigma through experiential learning. Fourth, in addition to addressing stigma, educational materials should also address practical barriers by clearly outlining referral processes, costs, and privacy protections, as the case of P10 demonstrates that even patients with a positive attitude may hesitate due to operational uncertainties. These strategies transform the observed coexistence of stigma and normalization into a phased, structured, and supportive approach to destigmatization tailored specifically for patients on chronic hemodialysis.

However, even patients who expressed a positive attitude toward seeking professional help during interviews admitted that they had never actually taken the first step to do so. What hinders them is no longer conceptual resistance, but practical obstacles at the operational level—such as uncertainty about which department to visit, concerns about being seen by acquaintances, lack of clarity regarding costs and reimbursement policies, and the transportation costs associated with traveling to and from the hospital. This finding suggests that a positive attitude alone is insufficient to prompt help-seeking behavior; practical constraints are equally significant barriers. While previous studies have largely focused on changing patients’ attitudes toward seeking help through health education (31, 32), this study indicates that future interventions must extend beyond attitude change to address practical barriers. Measures such as simplifying referral processes, clarifying cost policies, and ensuring privacy protection can help clear the obstacles to patients’ help-seeking behavior. Systemic solutions are essential: establishing clear referral processes to mental health professionals, providing remote mental health services that protect privacy, and clarifying reimbursement policies. These structural changes can turn intentions into action, thereby bridging the gap between intention and behavior.

### Practical implications for intervention and assessment

By translating the identified deficits in recognition, knowledge, and attitudes into targeted screening, stratified education, and system-level support, this study offers a conceptual pathway to enhance MHL and empower MHD patients in self-managing their mental health. The core significance of this study lies in providing an empirical foundation for the development of a mental health literacy scale specific to patients with MHD. The results of the qualitative interviews revealed unique characteristics of mental health literacy among patients with MHD—characteristics that are often difficult to fully capture in general-purpose mental health literacy scales.

Expressions mentioned by patients, such as “difficulty speaking openly” and “fear of being ridiculed,” can be directly translated into measurement items for the “stigma” dimension; patients’ limited understanding of professional help can be translated into items related to awareness of available resources within the “knowledge” dimension; and the cognitive disconnect between emotional perception and disease recognition observed in patients must be fully considered in the operational definition of “recognition ability.”

Based on the three themes and six subthemes identified in this study, future scale item pools should encompass core dimensions such as recognition ability, knowledge structure, and attitudinal orientation, thereby ensuring that the scale reflects both the general theoretical framework of mental health literacy and the specific characteristics of the MHD patient population.

### Cross-cultural comparisons and context-specific findings

Research has found that patients undergoing hemodialysis generally exhibit insufficient ability to recognize mental illness, fragmented knowledge of mental health, and significant barriers to seeking help due to stigma; these findings are largely consistent with those from other countries. Studies have amply demonstrated that dialysis patients endure severe psychological distress, yet this distress is often underrecognized and untreated; for example, among maintenance hemodialysis patients who exhibited symptoms of depression or anxiety and described barriers to mental health treatment, more than 70% failed to recognize their own symptoms or were unaware that they needed treatment (33). Research shows that the primary reason hemodialysis patients with depression fail to attend mental health clinics is the stigma associated with mental health treatment, including self-stigma, social stigma, and the burden of additional clinic visits (34), which resonates with the two subthemes we identified: stigma and concealment, and the gap between intention and action. Some studies have also pointed out a lack of comprehensive, structured mental health services within dialysis facilities; a qualitative study found that existing mental health support is both limited and informal, and lacks standardized screening, counseling, and referral pathways (35)—a finding consistent with our subthemes of insufficient awareness of services, barriers to access, and limited knowledge of professional resources. These commonalities suggest that the core dimensions of mental health literacy deficits are pervasive cross-cultural characteristics of the hemodialysis experience, rather than the product of specific contexts.

Nevertheless, the relative importance and manifestations of these barriers appear to be influenced by the local sociocultural and healthcare context. In Western settings, barriers such as cost, limited insurance coverage, and disparities in access to treatment are particularly prominent (36); whereas in a sample survey of rural areas in northwestern China, patients emphasized traditional perceptions of illness, reliance on informal social networks, and culturally rooted stigma and concealment—factors that may be further amplified in rural settings where resources and information are scarce. In China, the urban–rural divide is a particularly prominent factor affecting mental health literacy. A review found that patients living in rural areas, those with lower levels of education, those with lower household incomes, and those who are younger or male experience higher levels of stigma (37); rural residents’ limited access to information channels and relatively closed cultural environments were specifically identified as contributors to higher stigma (37). These findings are consistent with the subtheme of this study regarding limitations in awareness of professional resources, suggesting that the participants’ reliance on informal channels and limited awareness of professional mental health resources may, at least in part, reflect deficiencies specific to rural areas.

It is also notable that the prevalence of psychological disorders among Chinese MHD patients is reported to range from 24.75 to 50.56%, substantially higher than in the general chronic-disease population (35), and that psychological care interventions generally relieve anxiety and depression in this group (38, 39), underscoring the broader applicability of the need for targeted psychoeducation even beyond rural settings. Since most domestic studies to date have focused on quantitative surveys of the prevalence of anxiety and depression, the qualitative definition of the cognitive and attitudinal dimensions of mental health literacy provides complementary and generalizable research findings for a broader population with mental health disorders.

## Limitations

Several limitations should be acknowledged. ① Sampling and selection bias: All 11 participants were recruited from a single tertiary hospital in Ningxia. Although maximum diversity sampling was employed, the sample may not represent patients from other regions or private dialysis facilities. ② Generalizability: The environment in the Northwest is characterized by close-knit communities and limited mental health infrastructure, which may exacerbate certain manifestations, such as anxiety about disclosing information to roommates. It remains unclear to what extent these findings apply to populations in more economically developed regions. We therefore refrain from overgeneralizing, and recommend multicenter and cross-regional replication to test the stability and boundary conditions of these themes. ③ Researcher Positionality and Reflexivity: The research team comprised nurses with clinical and academic backgrounds in hemodialysis care. Although regular discussion meetings were held before and during data collection to document and acknowledge potential biases arising from these professional and academic orientations, the interviewers’ clinical perspective may have shaped both the phrasing of questions and the interpretation of participants’ accounts. While reflexivity was practiced through documented team discussions, we acknowledge that the positionality of the researchers cannot be entirely separated from the co-construction of the data, and that a team with different disciplinary or cultural standpoints might have elicited or emphasized somewhat different themes. ④ Interview Context: Data collection occurred within the hospital hemodialysis center, a clinical environment in which participants were actively receiving treatment. This setting may have constrained participants’ openness, particularly when discussing sensitive topics such as mental illness, stigma, and help-seeking reluctance. Moreover, interviews were conducted during a period of ongoing clinical care, which may have introduced fatigue or time pressure, potentially affecting the depth and candor of certain responses. Alternative settings or repeated interviews over time might have yielded richer accounts of participants’ shifting attitudes. ⑤ Sociodemographic subgroup differences: The sample size (*n* = 11) limits the ability to draw definitive conclusions regarding differences between subgroups defined by age, education, income, or digital literacy. While thematic patterns suggested stratification, formal subgroup comparisons were not possible; these observations should therefore be considered hypothesis-generating rather than conclusive.

Taken together, these limitations—rooted in the inherent nature of single-center qualitative inquiry—do not undermine the core value of the study, which lies in providing in-depth, contextually grounded accounts that complement existing quantitative surveys. However, they do highlight important directions for future research: multicenter and mixed-methods designs to verify transferability; triangulation through caregiver perspectives or standardized psychological measures to mitigate self-report and selection bias; explicit and documented reflexivity practices; and adequately powered studies to examine subgroup differences and to develop a culturally adapted mental health literacy scale for MHD patients.

## Conclusion

This study employed qualitative research methods and, based on O’Connor et al.’s simplified three-dimensional model of mental health literacy, conducted an in-depth exploration of the current status and characteristics of mental health literacy among patients with MHD. The study identified three core themes: individual differences in the ability to recognize mental illnesses, the multifaceted nature of mental health-related knowledge, and the dichotomous conflict between perceptions of mental illness and attitudes toward seeking help. The study found that MHD patients exhibit varying degrees of deficiencies across all dimensions of mental health literacy, with the most prominent challenges arising in the areas of mental illness recognition, acquisition of systematic knowledge, and overcoming the stigma associated with mental illness. Future research could be expanded in the following directions: conducting multicenter mixed-methods studies to verify the generalizability of the findings; developing and testing the reliability and validity of a mental health literacy scale for MHD patients based on the themes identified in this study; and developing and evaluating intervention strategies aimed at improving mental health literacy among MHD patients.

## Data Availability

The data analyzed in this study is subject to the following licenses/restrictions: due to ethical and privacy concerns, the raw interview data (audio recordings and verbatim transcripts) are not publicly available. All participants provided informed consent for the anonymized use of their data for research purposes only. To protect patient confidentiality and personal identity, the dataset is stored securely and can only be made available upon reasonable request to the corresponding author, subject to approval by the relevant Ethics Committee and a data use agreement. Requests to access these datasets should be directed to 1586418780@qq.com.
